# Supplementary material for: Policies on mental health in the workplace during the COVID-19 pandemic: A scoping review
Source: PLoS One. 2022 Jul 28;17(7):e0272296. doi: 10.1371/journal.pone.0272296 (PMC9333324; doi:10.1371/journal.pone.0272296)
Supplement: S1 File — (DOCX) [file pone.0272296.s001.docx]

**Supplementary material 1. Preferred Reporting Items for Systematic reviews and Meta-Analyses extension for Scoping Reviews (PRISMA-ScR) Checklist.**

| **SECTION** | **ITEM** | **PRISMA-ScR CHECKLIST ITEM** | **REPORTED ON PAGE #** |
| --- | --- | --- | --- |
| **TITLE** | | | |
| Title | 1 | Identify the report as a scoping review. | Page 1. |
| **ABSTRACT** | | | |
| Structured summary | 2 | Provide a structured summary that includes (as applicable): background, objectives, eligibility criteria, sources of evidence, charting methods, results, and conclusions that relate to the review questions and objectives. | Page 2. |
| **INTRODUCTION** | | | |
| Rationale | 3 | Describe the rationale for the review in the context of what is already known. Explain why the review questions/objectives lend themselves to a scoping review approach. | Background Section (page 3), paragraphs 67 to 96. |
| Objectives | 4 | Provide an explicit statement of the questions and objectives being addressed with reference to their key elements (e.g., population or participants, concepts, and context) or other relevant key elements used to conceptualize the review questions and/or objectives. | Background Section (page 3), paragraph 97 to 99. |
| **METHODS** | | | |
| Protocol and registration | 5 | Indicate whether a review protocol exists; state if and where it can be accessed (e.g., a Web address); and if available, provide registration information, including the registration number. | "Design and protocol" subsection (page 3), paragraph 105 to 106. |
| Eligibility criteria | 6 | Specify characteristics of the sources of evidence used as eligibility criteria (e.g., years considered, language, and publication status), and provide a rationale. | "Eligibility criteria" subsection (page 4), paragraph 121 to 125. |
| Information sources* | 7 | Describe all information sources in the search (e.g., databases with dates of coverage and contact with authors to identify additional sources), as well as the date the most recent search was executed. | “Search strategy” subsection (page 4), paragraph 113 to 115. |
| Search | 8 | Present the full electronic search strategy for at least 1 database, including any limits used, such that it could be repeated. | “Search strategy” subsection (page 3 and 4), paragraph 107 to 119. |
| Selection of sources of evidence† | 9 | State the process for selecting sources of evidence (i.e., screening and eligibility) included in the scoping review. | “Study selection” subsection (page 4), paragraph 126 to 142. |
| Data charting process‡ | 10 | Describe the methods of charting data from the included sources of evidence (e.g., calibrated forms or forms that have been tested by the team before their use, and whether data charting was done independently or in duplicate) and any processes for obtaining and confirming data from investigators. | “Study selection” subsection (page 4 and 5), paragraph 126 to 150. |
| Data items | 11 | List and define all variables for which data were sought and any assumptions and simplifications made. | “Data items” subsection (page 5), paragraph 151 to 159. |
| Critical appraisal of individual sources of evidence§ | 12 | If done, provide a rationale for conducting a critical appraisal of included sources of evidence; describe the methods used and how this information was used in any data synthesis (if appropriate). | Not applicable |
| Synthesis of results | 13 | Describe the methods of handling and summarizing the data that were charted. | “Synthesis of results” subsection (page 5), paragraph 160 to 168. |
| **RESULTS** | | | |
| Selection of sources of evidence | 14 | Give numbers of sources of evidence screened, assessed for eligibility, and included in the review, with reasons for exclusions at each stage, ideally using a flow diagram. | “Description the search” subsection (page 5), paragraph 170 to 176. |
| Characteristics of sources of evidence | 15 | For each source of evidence, present characteristics for which data were charted and provide the citations. | “Description the search” and “Excluded studies” subsections (page 5), paragraph 170 to 182. |
| Critical appraisal within sources of evidence | 16 | If done, present data on critical appraisal of included sources of evidence (see item 12). | Not applicable |
| Results of individual sources of evidence | 17 | For each included source of evidence, present the relevant data that were charted that relate to the review questions and objectives. | Not applicable |
| Synthesis of results | 18 | Summarize and/or present the charting results as they relate to the review questions and objectives. | “Included studies and Risk of bias” subsection (page 5 and 6), paragraph 183 to 185. |
| **DISCUSSION** | | | |
| Summary of evidence | 19 | Summarize the main results (including an overview of concepts, themes, and types of evidence available), link to the review questions and objectives, and consider the relevance to key groups. | Discussion Section (page 6 and 7), paragraph 187 to 258. |
| Limitations | 20 | Discuss the limitations of the scoping review process. | “Strengths and limitations” subsection (page 7 and 8), paragraph 259 to 271. |
| Conclusions | 21 | Provide a general interpretation of the results with respect to the review questions and objectives, as well as potential implications and/or next steps. | Conclusions Section (page 8), paragraph 272 to 279. |
| **FUNDING** | | | |
| Funding | 22 | Describe sources of funding for the included sources of evidence, as well as sources of funding for the scoping review. Describe the role of the funders of the scoping review. | “Funding” subsection (page 8), paragraph 291 to 296. |

JBI = Joanna Briggs Institute; PRISMA-ScR = Preferred Reporting Items for Systematic reviews and Meta-Analyses extension for Scoping Reviews.

* Where sources of evidence (see second footnote) are compiled from, such as bibliographic databases, social media platforms, and Web sites.

† A more inclusive/heterogeneous term used to account for the different types of evidence or data sources (e.g., quantitative and/or qualitative research, expert opinion, and policy documents) that may be eligible in a scoping review as opposed to only studies. This is not to be confused with information sources (see first footnote).

‡ The frameworks by Arksey and O’Malley (6) and Levac and colleagues (7) and the JBI guidance (4, 5) refer to the process of data extraction in a scoping review as data charting.

§ The process of systematically examining research evidence to assess its validity, results, and relevance before using it to inform a decision. This term is used for items 12 and 19 instead of "risk of bias" (which is more applicable to systematic reviews of interventions) to include and acknowledge the various sources of evidence that may be used in a scoping review (e.g., quantitative and/or qualitative research, expert opinion, and policy document).

**Supplementary material 2. Search strategies for each database.**

**Base:** Scopus

Search conducted October 14, 2021, at 12:00 pm (Peru time)

| No. | Search strategy | Records |
| --- | --- | --- |
| #1 | TITLE-ABS-KEY ("Mental Health" OR "Mental Disorders" OR "Mental problem*" OR "Mental Disorder" OR "Behavior Disorders" OR "Behavior Disorder" OR "Psychiatric disease" OR "Psychiatric diseases" OR "Psychiatric illness" OR "Psychiatric illnesses" OR "Psychiatric disorder" OR "Psychiatric disorders" OR "Mood disorder" OR "Mood disorders" OR "psychological problem" OR "psychological distress" OR "psychological stress*" OR "psychological disturb*" OR "psychological ill*" OR "psychological disease*" OR "emotional problem*" OR "emotional distress" OR "emotional stress*") | 735,114 |
| #2 | TITLE-ABS-KEY ("Depression" OR "Depressive Disorder" OR depress* OR "affective disorder" OR "affective disorders" OR "mood disorder" OR "mood disorders" OR dysphori* OR dysthymi* OR melancholia* OR anxiet* OR hypervigilance OR nervousness OR agoraphobia OR catastrophiz* OR phobi* OR panic* OR stress* OR distress* OR trauma* OR post-traumatic OR posttraumatic OR "Emotional Adjustment" OR "General disorder" OR "Emotional Adaptation" OR "Psychological Adjustment" OR "Psychologic Adaptation" OR "Adaptive Behavior" OR "Adjustment Disorder*") | 4,718,888 |
| #3 | TITLE-ABS ("COVID 19" OR "2019 novel coronavirus" OR "coronavirus" OR "coronavirus disease 2019" OR "coronaviridae" OR "2019-novel CoV" OR "2019 ncov" OR "ncov" OR "novel cov" OR "COVID 2019" OR "COVID19" OR "corona virus" OR "nCoV-2019" OR "nCoV2019" OR "nCoV 2019" OR "2019-ncov" OR "2019ncov" OR "COVID-19" OR "Severe acute respiratory syndrome coronavirus 2" OR "SARS-CoV-2" OR "SARS2" OR "SARS-CoV-19" OR "sars cov2") | 227,580 |
| #4 | TITLE-ABS-KEY (work-related OR work-load* OR workload* OR workplace* OR workplace* OR work* OR environment* OR "working condition" OR "working conditions" OR occupation* OR job OR jobs OR employ*) | 13,777,839 |
| #5 | TITLE-ABS-KEY ("national plan" OR "national program" OR "national strategy" OR legislation* OR law* OR "national reform" OR "health system" OR "Health Policy" OR "Health Policies" OR "Policy Implementation" OR "policy assessment" OR policy OR policies) | 2,971,909 |
| #6 | # 1 OR # 2 | 5,108,075 |
| #7 | # 3 AND # 4 AND # 5 AND # 6 | 1,483 |
| #8 | #7 AND ( LIMIT-TO ( PUBYEAR , 2022 ) OR LIMIT-TO ( PUBYEAR , 2021 ) OR LIMIT-TO ( PUBYEAR , 2020 ) ) | 1,480 |

**Base:** Pubmed

Search conducted October 14, 2021, at 12:00 pm (Peru time)

| No. | Search strategy | Records |
| --- | --- | --- |
| #1 | "Mental Health"[tiab] OR "Mental Disorders"[tiab] OR "Mental problem*"[tiab] OR "Mental Disorder"[tiab] OR "Behavior Disorders"[tiab] OR "Behavior Disorder"[tiab] OR "Psychiatric disease"[tiab] OR "Psychiatric diseases"[tiab] OR "Psychiatric illness"[tiab] OR "Psychiatric illnesses"[tiab] OR "Psychiatric disorder"[tiab] OR "Psychiatric disorders"[tiab] OR "Mood disorder"[tiab] OR "Mood disorders"[tiab] OR "psychological problem"[tiab] OR "psychological distress"[tiab] OR "psychological stress*"[tiab] OR "psychological disturb*"[tiab] OR "psychological ill*"[tiab] OR "psychological disease*"[tiab] OR "emotional problem*"[tiab] OR "emotional distress"[tiab] OR "emotional stress*"[tiab] OR "Mental Health"[mesh] OR "Mental Disorders"[mesh] OR "Mood Disorders"[mesh] OR "Stress, Psychological"[mesh] OR "Psychological Distress"[mesh] | 1,600,945 |
| #2 | "Depression"[tiab] OR "Depressive Disorder"[tiab] OR Depressi*[tiab] OR "affective disorder"[tiab] OR "affective disorders"[tiab] OR "mood disorder"[tiab] OR "mood disorders"[ tiab] OR dysphori*[tiab] OR dysthymi*[tiab] OR melancholia*[tiab] OR anxiet*[tiab] OR Hypervigilance[tiab] OR Nervousness[tiab] OR Agoraphobia[tiab] OR Catastrophiz*[tiab] OR Phobi*[tiab] OR Panic*[tiab] OR Stress*[tiab] OR Distress*[tiab] OR trauma*[tiab] OR Post-Traumatic[tiab] OR Posttraumatic[tiab] OR "Emotional Adjustment"[tiab] OR "General disorder"[tiab] OR "Emotional Adaptation"[tiab] OR "Psychological Adjustment"[tiab] OR "Psychologic Adaptation"[tiab] OR "Psychological Adaptation"[tiab] OR "Adaptive Behavior"[tiab] OR "Depression"[mesh] OR "Depressive Disorder"[mesh] OR "Anxiety Disorders"[mesh] OR "Anxiety"[mesh] OR "Agoraphobia"[mesh] OR "Phobic Disorders"[mesh] OR "Panic"[mesh] OR "Stress Disorders, Traumatic"[mesh] OR "Stress Disorders, Post-Traumatic"[mesh] OR "Stress, Psychological"[mesh] OR "Adaptation, Psychological"[mesh] OR "Psychological Distress"[mesh] OR "General Adaptation Syndrome"[mesh] OR "Adjustment Disorders"[mesh] OR "Stress, Psychological"[mesh] OR "Adaptation, Psychological"[mesh] OR "Psychological Distress"[mesh] OR "General Adaptation Syndrome"[mesh] OR "Adjustment Disorders"[mesh] | 2,103,607 |
| #3 | "coronavirus"[MeSH] OR "coronavirus infections"[MeSH] OR "coronavirus"[All Fields] OR "covid 2019"[All Fields] OR "SARS2"[All Fields] OR "SARS-CoV-2"[All Fields] OR "SARS-CoV-19"[All Fields] OR "coronavirus infection"[All Fields] OR "novel cov"[All Fields] OR "2019ncov"[ All Fields] OR "sars cov2"[All Fields] OR "ncov"[All Fields] OR "covid-19"[All Fields] OR "covid19"[All Fields] OR "coronaviridae"[All Fields] OR "corona virus"[All Fields] | 205,191 |
| #4 | "Work"[MeSH] OR "Workload"[Mesh] OR "Workplace"[Mesh] OR "Occupational Health"[Mesh] OR "Occupational Diseases"[Mesh] OR "Occupational Groups"[Mesh] OR "Occupational Exposure"[Mesh] OR "Occupations"[Mesh] OR "Women, Working"[Mesh] OR "Employment"[Mesh: NoExp] OR work-related[tiab] OR work-load*[tiab] OR workload*[tiab] OR workplace*[tiab] OR workplace*[tiab] OR work*[tiab] OR environment*[tiab] OR "working condition"[tiab] OR "working conditions"[tiab] OR occupation*[tiab] OR job[tiab] OR jobs[tiab] OR employ*[tiab] | 3,900,187 |
| #5 | "national plan"[tiab] OR "national program"[tiab] OR "national strategy"[tiab] OR legislation*[tiab] OR law[tiab] OR "national reform"[tiab] OR "health system"[tiab] OR "Health Policy"[tiab] OR "Health Policies"[tiab] OR "Policy Implementation"[tiab] OR "policy assessment"[tiab] OR policy[tiab] OR policies[tiab] | 454,690 |
| #6 | #1 OR #2 | 3,424,244 |
| #7 | #3 AND #4 AND #5 AND #6 | 1,005 |
| #8 | #7 limit 2022 OR 2020 OR 2021 | 988 |

**Base:** Web of Science

Search conducted October 14, 2021, at 12:00 pm (Peru time)

| No. | Search strategy | Records |
| --- | --- | --- |
| #1 | TS = ("Mental Health" OR "Mental Disorders" OR "Mental problem*" OR "Mental Disorder" OR "Behavior Disorders" OR "Behavior Disorder" OR "Psychiatric disease" OR "Psychiatric diseases" OR "Psychiatric illness" OR "Psychiatric illnesses" OR "Psychiatric disorder" OR "Psychiatric disorders" OR "Mood disorder" OR "Mood disorders" OR "psychological problem" OR "psychological distress" OR "psychological stress*" OR "psychological disturb*" OR "psychological ill*" OR "psychological disease*" OR "emotional problem*" OR "emotional distress" OR "emotional stress*") | 410,628 |
| #2 | TS = ("Depression" OR "Depressive Disorder" OR depress* OR "affective disorder" OR "affective disorders" OR "mood disorder" OR "mood disorders" OR dysphori* OR dysthymi* OR melancholia* OR anxiet* OR hypervigilance OR nervousness OR agoraphobia OR catastrophiz* OR phobi* OR panic* OR stress* OR distress* OR trauma* OR post-traumatic OR posttraumatic OR "Emotional Adjustment" OR "General disorder" OR "Emotional Adaptation" OR "Psychological Adjustment" OR "Psychologic Adaptation" OR "Adaptive Behavior" OR "Adjustment Disorder*") | 3,114,074 |
| #3 | TS = ("COVID 19" OR "2019 novel coronavirus" OR "coronavirus" OR "coronavirus disease 2019" OR "coronaviridae" OR "2019-novel CoV" OR "2019 ncov" OR "ncov" OR "novel cov" OR "COVID 2019" OR "COVID19" OR "corona virus" OR "nCoV-2019" OR "nCoV2019" OR "nCoV 2019" OR "2019-ncov" OR "2019ncov" OR "COVID-19" OR "Severe acute respiratory syndrome coronavirus 2" OR "SARS-CoV-2" OR "SARS2" OR "SARS-CoV-19" OR "sars cov2") | 209,150 |
| #4 | TS = (work-related OR "work-load"* OR workload* OR workplace* OR workplace* OR work* OR environment* OR "working condition" OR "working conditions" OR occupation* OR job OR jobs OR employ*) | 7,411,472 |
| #5 | TS = ("national plan" OR "national program" OR "national strategy" OR legislation* OR law* OR "national reform" OR "health system" OR "Health Policy" OR "Health Policies" OR "Policy Implementation" OR "policy assessment" OR policy OR policies) | 1,575,945 |
| #6 | #1 OR # 2 | 3,282,031 |
| #7 | #3 AND #4 AND #5 AND #6 | 1,021 |
| #8 | #7 AND (2022 or 2021 or 2020 (Publication Years)) | 1,017 |

**Base:** EMBASE

Search conducted March 22, 2021, at 3:00 pm (Peru time)

| No. | Search strategy | Records |
| --- | --- | --- |
| #1 | 'mental health':ti,ab,kw OR 'mental disorders':ti,ab,kw OR 'mental problem*':ti,ab,kw OR 'mental disorder':ti,ab,kw OR 'behavior disorders':ti,ab,kw OR 'behavior disorder':ti,ab,kw OR 'psychiatric disease':ti,ab,kw OR 'psychiatric diseases':ti,ab,kw OR 'psychiatric illness':ti,ab,kw OR 'psychiatric illnesses':ti,ab,kw OR 'psychiatric disorder':ti,ab,kw OR 'psychiatric disorders':ti,ab,kw OR 'psychological problem':ti,ab,kw OR 'psychological distress':ti,ab,kw OR 'psychological stress*':ti,ab,kw OR 'psychological disturb*':ti,ab,kw OR 'psychological ill*':ti,ab,kw OR 'psychological disease*':ti,ab,kw OR 'emotional problem*':ti,ab,kw OR 'emotional distress':ti,ab,kw OR 'emotional stress*':ti,ab,kw OR 'depression':ti,ab,kw OR 'depressive disorder':ti,ab,kw OR depress*:ti,ab,kw OR 'affective disorder':ti,ab,kw OR 'affective disorders':ti,ab,kw OR 'mood disorder':ti,ab,kw OR 'mood disorders':ti,ab,kw OR dysphori*:ti,ab,kw OR dysthymi*:ti,ab,kw OR melancholia*:ti,ab,kw OR anxiet*:ti,ab,kw OR hypervigilance:ti,ab,kw OR nervousness:ti,ab,kw OR agoraphobia:ti,ab,kw OR catastrophiz*:ti,ab,kw OR phobi*:ti,ab,kw OR panic*:ti,ab,kw OR stress*:ti,ab,kw OR distress*:ti,ab,kw OR trauma*:ti,ab,kw OR 'post traumatic':ti,ab,kw OR posttraumatic:ti,ab,kw OR 'emotional adjustment':ti,ab,kw OR 'general disorder':ti,ab,kw OR 'emotional adaptation':ti,ab,kw OR 'psychological adjustment':ti,ab,kw OR 'psychologic adaptation':ti,ab,kw OR 'adaptive behavior':ti,ab,kw OR 'adjustment disorder*':ti,ab,kw | 2,782,179 |
| #2 | 'covid 19':ti,ab,kw OR '2019 novel coronavirus':ti,ab,kw OR 'coronavirus':ti,ab,kw OR 'coronavirus disease 2019':ti,ab,kw OR 'coronaviridae':ti,ab,kw OR '2019-novel cov':ti,ab,kw OR '2019 ncov':ti,ab,kw OR 'ncov':ti,ab,kw OR 'novel cov':ti,ab,kw OR 'covid 2019':ti,ab,kw OR 'covid19':ti,ab,kw OR 'corona virus':ti,ab,kw OR 'ncov-2019':ti,ab,kw OR 'ncov2019':ti,ab,kw OR 'ncov 2019':ti,ab,kw OR '2019-ncov':ti,ab,kw OR '2019ncov':ti,ab,kw OR 'covid-19':ti,ab,kw OR 'severe acute respiratory syndrome coronavirus 2':ti,ab,kw OR 'sars-cov-2':ti,ab,kw OR 'sars2':ti,ab,kw OR 'sars-cov-19':ti,ab,kw OR 'sars cov2':ti,ab,kw | 195,677 |
| #3 | 'work related':ti,ab,kw OR 'work load*':ti,ab,kw OR workload*:ti,ab,kw OR workplace*:ti,ab,kw OR work*:ti,ab,kw OR environment*:ti,ab,kw OR 'working condition':ti,ab,kw OR 'working conditions':ti,ab,kw OR occupation*:ti,ab,kw OR job:ti,ab,kw OR jobs:ti,ab,kw OR employ*:ti,ab,kw | 4,104,749 |
| #4 | 'national plan':ti,ab,kw OR 'national program':ti,ab,kw OR 'national strategy':ti,ab,kw OR legislation*:ti,ab,kw OR law*:ti,ab,kw OR 'national reform':ti,ab,kw OR 'health system':ti,ab,kw OR 'health policy':ti,ab,kw OR 'health policies':ti,ab,kw OR 'policy implementation':ti,ab,kw OR 'policy assessment':ti,ab,kw OR policy:ti,ab,kw OR policies:ti,ab,kw | 598,013 |
| #5 | #1 AND #2 AND #3 AND #4 | 779 |
| #6 | #5 AND (2020:py OR 2021:py OR 2022:py) | 776 |

**Supplementary material 3. Reasons for exclusion of documents that were submitted to full-text revision (n=43).**

| Title | Year | Journal | Authors | Decision |
| --- | --- | --- | --- | --- |
| 1. Essential and Vulnerable: Implications of Covid-19 for Farmers in Ireland | 2020 | J Agro\|medicine | Meredith, D. and McNamara, J. and van Doorn, D. and Richardson, N. | Excluded: Does not evaluate a mental health policy in occupational groups. |
| 1. Emotional distress, psychosomatic symptoms and their relationship with institutional responses: A survey of Italian frontline medical staff during the Covid-19 pandemic | 2020 | Heliyon | Marinaci, T. and Carpinelli, L. and Venuleo, C. and Savarese, G. and Cavallo, P. | Excluded: Does not evaluate a mental health policy in occupational groups. |
| 1. The effect of COVID-19 pandemic on the mental health of Canadian critical care nurses providing patient care during the early phase pandemic: A mixed method study | 2020 | Intensive and Critical Care Nursing | Crowe, S. and Howard, AF and V and erspank-Wright, B. and Gillis, P. and McLeod, F. and Penner, C. and Haljan, G. | Excluded: Does not evaluate a mental health policy in occupational groups. |
| 1. Educational Inequalities Derived from Covid-19 from a Feminist Perspective. Analysis of the Discourse of Madrid Education Professionals | 2020 | International Journal of Education for Social Justice | Tosso, MP and Sainz, MS and Casado, CM | Excluded: Does not evaluate a mental health policy in occupational groups. |
| 1. Easing the disruption of COVID-19: supporting the mental health of the people of Canada-October 2020-an RSC Policy Briefing | 2020 | Facets | Asmundson, GJG and Blackstock, C. and Bourque, MC and Brimacombe, G. and Crawford, A. and Deacon, SH and McMullen, K. and McGrath, PJ and Mushquash, C. and Stewart, SH and Stinson, J. and Taylor, S. and Campbell-Yeo, M. | Excluded: Does not evaluate a mental health policy in occupational groups. |
| 1. Development of wellness programs during the COVID-19 pandemic response | 2020 | Psychiatric Annals | Spray, AM and Patel, NA and Sood, A. and Wu, SX and Simon, NM and Podbury, R. and Vasserman, A. and Caravella, RA and Silverman, Y. and Pochtar, R. and Liaw, KRL and Ackerman , MG | Excluded: Institutional intervention, not a policy. |
| 1. COVID-19: Korean nurses 'experiences and ongoing tasks for the pandemic' s second wave | 2020 | International Nursing Review | Kang, Y. and Shin, KR | Excluded: Does not evaluate a mental health policy in occupational groups. |
| 1. COVID-19, health rights of prison staff, and the bridge between prison and public health in Africa | 2020 | Public Health | Van Hout, MC | Excluded: It is a letter to the editor. |
| 1. The COVID-19 Pandemic and Italian Public Mental Health Services: Experience and Future Directions | 2020 | J Patient Exp | Pelizza, L. and Pupo, S. | Excluded: Does not evaluate a mental health policy in occupational groups. |
| 1. The COVID-19 Pandemic and Internal Labor Migration in India: A 'Crisis of Mobility' | 2020 | Indian Journal of Labor Economics | Irudaya Rajan, S. and Sivakumar, P. and Srinivasan, A. | Excluded: Does not evaluate a mental health policy in occupational groups. |
| 1. COVID-19 lockdown: India struggles to feed migrants left behind | 2021 | Asia Pacific Journal of Social Work and Development | Shahare, VB | Excluded: Does not evaluate a mental health policy in occupational groups. |
| 1. COVID-19 Lessons: The Alignment of Palliative Medicine and Trauma-Informed Care | 2020 | Journal of Pain and Symptom Management | Brown, C. and Peck, S. and Humphreys, J. and Schoenherr, L. and Saks, NT and Sumser, B. and Elia, G. | Excluded: Does not evaluate a mental health policy in occupational groups. |
| 1. COVID-19 and the Correctional Environment: The American Prison as a Focal Point for Public Health | 2020 | American Journal of Preventive Medicine | Montoya-Barthelemy, AG and Lee, CD and Cundiff, DR and Smith, EB | Excluded: Does not evaluate a mental health policy in occupational groups. |
| 1. Coping With Trauma, Celebrating Life: Reinventing Patient And Staff Support During The COVID-19 Pandemic | 2020 | Health Aff (Millwood) | Wei, E. and Segall, J. and Villanueva, Y. and Dang, LB and Gasca, VI and Gonzalez, MP and Roman, M. and Mendez-Justiniano, I. and Cohen, AG and Cho, HJ | Excluded: Does not evaluate a mental health policy in occupational groups. |
| 1. A Case Study of the Westchester County New York's Jail Response to COVID-19: Controlling COVID while Balancing Service Needs for the Incarcerated-A National Model for Jails | 2020 | Victims and Offenders | Collica-Cox, K. and Molina, L. | Excluded: Does not evaluate a mental health policy in occupational groups. |
| 1. Mental Health Community and Health System Issues in COVID-19: Lessons from Academic, Community, Provider and Policy Stakeholders | 2020 | Ethnicity & disease | Arevian, AC and Jones, F. and Moore, EM and Goodsmith, N. and Aguilar-Gaxiola, S. and Ewing, T. and Siddiq, H. and Lester, P. and Cheung, E. and Ijadi-Maghsoodi, R . and Gabrielian, S. and Sugarman, OK and Bonds, C. and Benitez, C. and Innes-Gomberg, D. and Springgate, B. and Haywood, C. and Meyers, D. and Sherin, JE and Wells, K. | Excluded: Does not evaluate a mental health policy in occupational groups. |
| 1. Mental Health And Holistic Care of Migrant Workers in Singapore During the Covid-19 Pandemic | 2020 | Journal of Global Health | Chan, LG and Kuan, B. | Excluded: Does not evaluate a mental health policy in occupational groups. |
| 1. The impact of COVID-19 on allied health professions | 2020 | PLoS ONE | Coto, J. and Restrepo, A. and Cejas, I. and Prentiss, S. | Excluded: Does not evaluate a mental health policy in occupational groups. |
| 1. Experiences and Psychological Adjustments of Nurses Who Voluntarily Supported COVID-19 Patients in Hubei Province, China | 2020 | Psychology Research and Behavior Management | Cui, SS and Zhang, L. and Yan, HY and Shi, QY and Jiang, YJ and Wang, Q. and Chu, J. | Excluded: Does not evaluate a mental health policy in occupational groups. |
| 1. How do state policies shape experiences of household income shocks and mental health during the COVID-19 pandemic? | 2021 | Social Science and Medicine | Donnelly, R. and Farina, MP | Excluded: Does not evaluate a mental health policy in occupational groups. |
| 1. Mental health and capacity laws in Northern Ireland and the COVID-19 pandemic: Examining powers, procedures and protections under emergency legislation | 2020 | International Journal of Law and Psychiatry | Farrell, AM and Hann, P. | Excluded: Does not evaluate a mental health policy in occupational groups. |
| 1. Nurses' Workplace Conditions Impacting Their Mental Health during COVID-19: A Cross-Sectional Survey Study | 2021 | Healthcare (Basel) | Havaei, F. and Ma, A. and Staempfli, S. and MacPhee, M. | Excluded: Does not evaluate a mental health policy in occupational groups. |
| 1. Mobilizing an institutional supportive response for healthcare workers and other staff in the context of COVID-19: The Yale experience | 2021 | General Hospital Psychiatry | Krystal, JH and Alvarado, J. and Ball, SA and Fortunati, FG and Hu, M. and Ivy, ME and Kapo, J. and Olson, KD and Rohrbaugh, RM and Sinha, R. and Tebes, JK and Vender, RJ and Yonkers, KA and Mayes, LC | Excluded: Does not evaluate a mental health policy in occupational groups. |
| 1. Future psychiatric services in Italy: Lesson from the COVID-19 pandemic | 2020 | Biomedical Act | Pelizza, L. and Pupo, S. | Excluded: Does not evaluate a mental health policy in occupational groups. |
| 1. The implications of COVID-19 for health workforce planning and policy: the case of Peru | 2021 | The International journal of health planning and management | Rees, GH and Peralta Quispe, F. and Scotter, C. | Excluded: Does not evaluate a mental health policy in occupational groups. |
| 1. Policing 'a pandemic: Garda wellbeing and COVID-19 | 2020 | Irish Journal of Psychological Medicine | Rooney, L. and McNicholas, F. | Excluded: Editorial-type comments and recommendations. |
| 1. Preventing a Parallel Pandemic - A National Strategy to Protect Clinicians' Well-Being | 2020 | N Engl J Med | Dzau, VJ and Kirch, D. and Nasca, T. | Excluded: Editorial-type comments and recommendations. |
| 1. A study of coping stress strategies on psychological well-being during the COVID-19 pandemic in Jabodetabek Area | 2020 | 2020 International Conference on ICT for Smart Society (ICISS) | Sofa, GA and Findez Shidiq Anugrah, A. and Nugraha, Y. and Al Rasyid, SH and Aghinasuci, V. and Hidayat, WN and Wibowo, I. and Suherman, AL | Excluded: Does not evaluate a mental health policy in occupational groups. |
| 1. Perceived sources of occupational burn-out and embitterment among front-line health workers for COVID-19 control in Gyeonggi province, South Korea: a qualitative study | 2021 | Occupational and Environmental Medicine | Kang B, Kwon S, You M, Lee H | Excluded: Does not evaluate a mental health policy in occupational groups. |
| 1. An Intervention to Increase Situational Awareness and the Culture of Mutual Care (Foco) and Its Effects During COVID-19 Pandemic: A Randomized Controlled Trial and Qualitative Analysis | 2020 | Frontiers in Psychiatry | Kozasa E H, Lacerda S S, Polissici M A, Coelho R D S, Farias G D S, Chaves P, Leão E R | Excluded: Does not evaluate a mental health policy in occupational groups. |
| 1. Subjective burden and perspectives of German healthcare workers during the COVID-19 pandemic | 2021 | European Archives of Psychiatry and Clinical Neuroscience | Kramer V, Papazova I, Thoma A, Kunz M, Falkai P, Schneider-Axmann T, Hierundar A, Wagner E, Hasan A | Excluded: Does not evaluate a mental health policy in occupational groups. |
| 1. Public trust and media influence on anxiety and depression levels among skilled workers during the COVID-19 outbreak in Serbia | 2020 | Vojnosanitetski pregled | Marković I, Nikolovski S, Milojević S, Živković D, Knežević S, Mitrović A, Fišer Z, Djurdjević D | Excluded: Does not evaluate a mental health policy in occupational groups. |
| 1. CE: Providing Care for Caregivers During COVID-19 | 2021 | American Journal of Nursing | Morales C, Brown M | Excluded: Does not evaluate a mental health policy in occupational groups. |
| 1. "We Are Saving Their Bodies and Destroying Their Souls.": Family Caregivers' Experiences of Formal Care Setting Visitation Restrictions during the COVID-19 Pandemic | 2021 | Journal of Aging & Social Policy | Nash W A, Harris L M, Heller K E, Mitchell B D | Excluded: Does not evaluate a mental health policy in occupational groups. |
| 1. Characteristics of peer respites in the United States: Expanding the continuum of care for psychiatric crisis | 2021 | Psychiatric Rehabilitation Journal | Pelot M, Ostrow L | Excluded: Does not evaluate a mental health policy in occupational groups. |
| 1. Psychological impact of the COVID-19 pandemic within institutional quarantine and isolation centres and its sociodemographic correlates in Qatar: a cross-sectional study | 2021 | BMJ Open | Reagu, S.; Wadoo, O.; Latoo, J.; Nelson, D.; Ouanes, S.; Masoodi, N.; Karim, M. A.; Iqbal, Y.; Al Abdulla, S.; Al Nuaimi, S. K.; Abdelmajid, A. A. B.; Al Samawi, M. S.; Khoodoruth, M. A. S.; Khoodoruth, W. N. C.; Al-Maslamani, Mars; Alabdulla, M | Excluded: Does not evaluate a mental health policy in occupational groups. |
| 1. Psychological impact of the lockdown due to the covid-19 pandemic in university workers: Factors related to stress, anxiety, and depression | 2021 | International Journal of Environmental Research and Public Health | Salazar, A.; Palomo-Osuna, J.; de Sola, H.; Moral-Munoz, J. A.; Dueñas, M.; Failde, I. | Excluded: Does not evaluate a mental health policy in occupational groups. |
| 1. Vaccination willingness against COVID-19 among healthcare workers in Germany: Results from a University Medicine Network survey between November 2020 and January 2021 | 2021 | Bundesgesundheitsblatt - Gesundheitsforschung - Gesundheitsschutz | Schug, C.; Erim, Y.; Geiser, F.; Hiebel, N.; Beschoner, P.; Jerg-Bretzke, L.; Albus, C.; Weidner, K.; Steudte-Schmiedgen, S.; Borho, A.; Lieb, M.; Morawa, E. | Excluded: Does not evaluate a mental health policy in occupational groups. |
| 1. Burnout among healthcare providers during COVID-19: Challenges and evidence-based interventions | 2020 | Indian Journal of Medical Ethics | Sultana, A.; Sharma, R.; Hossain, M. M.; Bhattacharya, S.; Purohit, N. | Excluded: Does not evaluate a mental health policy |
| 1. Psychological Impacts and Post-Traumatic Stress Disorder among People under COVID-19 Quarantine and Isolation: A Global Survey | 2021 | International Journal of Environmental Research and Public Health | TMGH-Global COVID-19 Collaborative, et al. | Excluded: Does not evaluate a mental health policy in occupational groups. |
| 1. Solutions to prevent and address physician burnout during the pandemic in Mexico | 2020 | Indian Journal of Psychiatry | Ng, B. | Excluded: Institutional intervention, not a policy |
| 1. Community interventions for improving adult mental health: mapping local policy and practice in England | 2021 | BMC Public Health | F Duncan, C Baskin, M McGrath, J F Coker, C Lee, J Dykxhoorn, E A Adams, S Gnani, L Lafortune, J B Kirkbride, E Kaner, O Jones, G Samuel, K Walters, D Osborn, E J Oliver | Excluded: Includes studies prior to the COVID-19 pandemic. |
| 1. China’s Mental Health Interventions During the COVID-19 Outbreak | 2020 | Psychology in Russia: State of the Art | Zhengkui Liu, Yuanyuan An, Kankan Wua, | Excluded: Institutional intervention, not a policy |
