# Supplementary material for: Policies on mental health in the workplace during the COVID-19 pandemic: A scoping review
Source: PLoS One. 2022 Jul 28;17(7):e0272296. doi: 10.1371/journal.pone.0272296 (PMC9333324; doi:10.1371/journal.pone.0272296)
Supplement: Attachment — Submitted filename: 3. Responce letter.docx [file pone.0272296.s002.docx]

December 2021

Dear Editor,

We would like to thank you and the reviewers for your comments. We have carefully reviewed them and have revised the manuscript accordingly. Our responses are detailed below. Highlighted in red are the changes to the manuscript. We hope the revised version is now suitable for publication and look forward to hearing from you in due course.

Sincerely,

The authors,

-------

I feel that your manuscript deals with an important topic and would like to encourage you to resubmit your paper to PLOS ONE, but in the light of the unparalleled speed of research in terms of the COVID-pandemic, your scoping review is in need of an update to avoid being outdated even at the time of its formal publication.

Reply: We have performed the search again in October and submit this new version of the manuscript for your consideration. Various modifications are made to the text in the results and discussion section.

I redid your literature search for Web of Science using your approach and obtained several potentially relevant hits that have been published this summer. (e.g., Shields et al., 2021, Social Psychiatry and Psychiatric Epidemiology; Cai et al., 2021; World Journal of Psychiatry) and which may therefore likely substantially alter the conclusions of your ms. If you are willing to update your review, I would be happy to reconsider your paper as a new submission.

Reply: When we searched again we found one study that was included in the scoping review. We have reviewed the recommended papers and found that they should not be entered.

Shields et al., 2021, Social Psychiatry and Psychiatric Epidemiology = It does not focus on policy evaluation in the context of COVID-19.

Cai et al., 2021; World Journal of Psychiatry = It is a narrative review, so it does not meet the requirement of using primary or secondary data.
