# Supplementary material for: Policies on mental health in the workplace during the COVID-19 pandemic: A scoping review
Source: PLoS One. 2022 Jul 28;17(7):e0272296. doi: 10.1371/journal.pone.0272296 (PMC9333324; doi:10.1371/journal.pone.0272296)
Supplement: Attachment — Submitted filename: responses.docx [file pone.0272296.s003.docx]

- Your eligibility includes literature that “addressed national policies promoted by official governments, that were in execution or that had been executed, and that were directly or indirectly related to mental health in the workplace” - since the scope is primarily national policies, I would suggest updating your title to include this

- If the goal was to identify government implemented policies, wondering if you had considered including a grey literature search within the review, as certain policy-related evaluations might have not made their way to evidence based databases (e.g. Scopus/ Web of Science/ Embase and Pub Med)?

- Keeping in mind that scoping reviews aim to “map the literature on a particular topic or research area and provide an opportunity to identify key concepts; gaps in the research; and types and sources of evidence to inform practice, policymaking, and research” (Daudt et al. 2013) - since there was only a single paper included within your report, it would be worth it to do an additional synthesis of the full-text review articles that focused on national COVID-19 mental health policies (even through they did not evaluate outcomes), to truly describe the gaps in research and provide the reader with a thorough synthesis of the literature - using data from Supplementary Table 3 & 4.

- The authors outline two important limitations of it being too early to evaluate policies OR that mental health outcomes may be evaluated in larger COVID-19 strategies (instead of MH specific ones), which were also points that I had while reading through the manuscript - it could be worth to re-do the search in a year or two.

- In certain countries like Canada, where healthcare is delivered on a provincial basis, there were governmental provincial policies rather than national policies implemented, so perhaps including the justification to only focus on national policies within your limitations would be appropriate.

- The paper would benefit from a thorough read through for grammar:  E.g. “The study protocol in PROSPERO, since this repository does not support scoping review.”
